# Physician-level variation in the diagnosis of myocardial infarction and the use of angiography among Veterans with elevated troponin

**DOI:** 10.1186/s40779-016-0090-5

**Published:** 2016-07-25

**Authors:** David E. Winchester, Nayan Agarwal, Lucas Burke, Steven Bradley, Tatiana Schember, Carsten Schmalfuss

**Affiliations:** Division of Cardiovascular Medicine, University of Florida College of Medicine, Gainesville, FL USA; Malcom Randall Veterans Affairs Medical Center, Gainesville, FL USA; Division of Cardiology, Department of Medicine, VA Eastern Colorado Health Care System, Denver, CO USA; Department of Medicine, University of Colorado School of Medicine at the Anschutz Medical Campus, Aurora, CO USA

**Keywords:** Acute coronary syndrome, Coronary angiography, Variation in care, Cardiac troponin

## Abstract

**Background:**

Cardiac troponin assays have improved the ability to detect myocardial damage. However, ascertaining whether troponin elevation is due to myocardial infarction (MI) or secondary to another process can be challenging. Our aim is to evaluate provider-level variation in the diagnosis of MI and the use of invasive coronary angiography (ICA) among patients with undifferentiated elevations in cardiac troponin.

**Methods:**

We analyzed data from all patients with elevated troponin levels in a single Veterans Affairs (VA) Medical Center between 2006 and 2007. One of several cardiologists prospectively evaluated each patient’s presentation and course of care. We compared the frequency of MI diagnosis and ICA use between physicians using univariate odds ratios (*OR*).

**Results:**

Among 761 patients, 34.0 % were diagnosed with MI and 25.9 % underwent ICA. The unadjusted rates of MI (23.9 to 56.7 %, *P* = 0.02) and ICA (17.3 to 73.3 %, *P* < 0.001) differed between physicians. Comparing the patient cohorts for each physician, baseline characteristics were similar except for chest pain. In multivariate regression, factors associated with the use of cardiac ICA included an abnormal electrocardiograph (ECG) (*OR* = 1.89, *P* = 0.014), level of troponin (*OR* = 1.71, *P* = 0.004), chest pain (*OR* = 8.60, *P* < 0.001), and care by non-VA physicians (*OR* = 4.45, *P* = 0.006). One physician had a lower ICA use (*OR* = 0.56, *P* = 0.017). In multivariate regression of MI, no physician-level variation was observed.

**Conclusion:**

Among patients with elevated troponin, the likelihood of being diagnosed with MI and undergoing ICA is dependent on their clinical presentation. After adjustment, physician-level variation in care was observed for the use of ICA, but not for the diagnosis of MI.

## Background

Cardiac troponin (Tn) is a sensitive marker of myocardial necrosis [1], but Tn can be elevated in many disease states without clinical evidence of myocardial infarction (MI) [2, 3]. The universal definition of myocardial infarction is a rise and/or fall of a cardiac biomarker, preferably Tn, in addition to a clinical presentation consistent with MI [4]. Despite this, physicians may disagree when applying this definition to individual patients. Accurate clinical diagnosis of MI is important to guide appropriate utilization of diagnostic procedures and risk modifying therapies.

Variation in the use of medical testing and the interpretation of a diagnostic test is well documented in the medical literature and extends to the level of the individual provider [5, 6]. The use of cardiac testing and the interpretation of cardiac tests is also subject to significant variation [7–9]. Indeed, there is approximately a 2-fold regional variation in the use of invasive coronary angiography (ICA) after acute myocardial infarction within the United States [10, 11].

As part of a local quality improvement effort, our facility tracked all patients with elevated Tn for a one-year period. This provided a unique opportunity to evaluate patterns of MI diagnosis and the use of ICA in response to elevated Tn. We analyzed that database to determine if the care of patients with elevated Tn differed among providers.

## Methods

We conducted a single center retrospective cohort study at our Veterans Affairs (VA) Medical Center. Data were obtained from a quality improvement program conducted on patients with elevated Tn who were seen in our facility between February 2006 and February 2007. The study protocol was reviewed by the Institutional Review Board-01 Gainesville Health Science Center, and the requirement for informed consent was waived. During that time frame, a “troponin team” evaluated every patient with an elevated Tn, defined as being greater than 0.03 ng/ml on our Tn-T assay. The team was led by a clinical coordinator who received a daily list from the facility’s core laboratory of all patients who had an elevated Tn. This coordinator evaluated each patient based on their chart documentation and presented all patients to a cardiologist who determined if the patient’s presentation was consistent with a MI and whether ICA should be performed. No formal definition of MI was applied, and physicians were free to make the diagnosis based on their clinical assessment of the patient. Because our facility does not have an inpatient service led by a cardiologist, all other care decisions were left to the admitting team with input from the cardiology consultation service. Several cardiologists shared responsibility for oversight of the “troponin team” including general/noninvasive, interventional, and electrophysiology cardiologists. Data were blinded as to the physician, and therefore could not be analyzed at the level of subspecialty within cardiology. Data, including each patient’s baseline clinical characteristics, clinical course, thrombolysis in myocardial infarction (TIMI) score, electrocardiogram (ECG) results, and follow-up, were recorded by the clinical coordinator.

The co-primary outcomes were the rates of MI diagnosis and ICA use between individual physicians, which were compared using chi square analysis. We conducted a multivariate logistic regression to determine how the diagnosis of MI and the use of ICA were affected by the following variables: age by year, sex, coronary artery disease, hypertension, diabetes mellitus, hyperlipidemia, prior coronary intervention, prior coronary bypass surgery, ECG changes, new ECG changes, primary symptom of chest pain / dyspnea / other, serum creatinine, level of 1st Tn measurement. Each physician was included as an independent variable with the remainder of the physicians for comparison. We considered attempting an additional hierarchical multi-level model to compare odds ratios for MI diagnosis and ICA use between physicians after accounting for clustering of patients within their physician-based cohort. Due to the small sample sizes, however, this model was not considered statistically valid [12–14]. Odds ratios (*OR*) and 95 % confidence intervals (CI) were reported. Because we used an existing dataset, no formal a priori power calculation was performed. Baseline variables were compared by parametric and nonparametric tests as appropriate. Analysis was performed using SPSS version 21 (IBM, Armonk, NY).

## Results

**Fig. 1:**
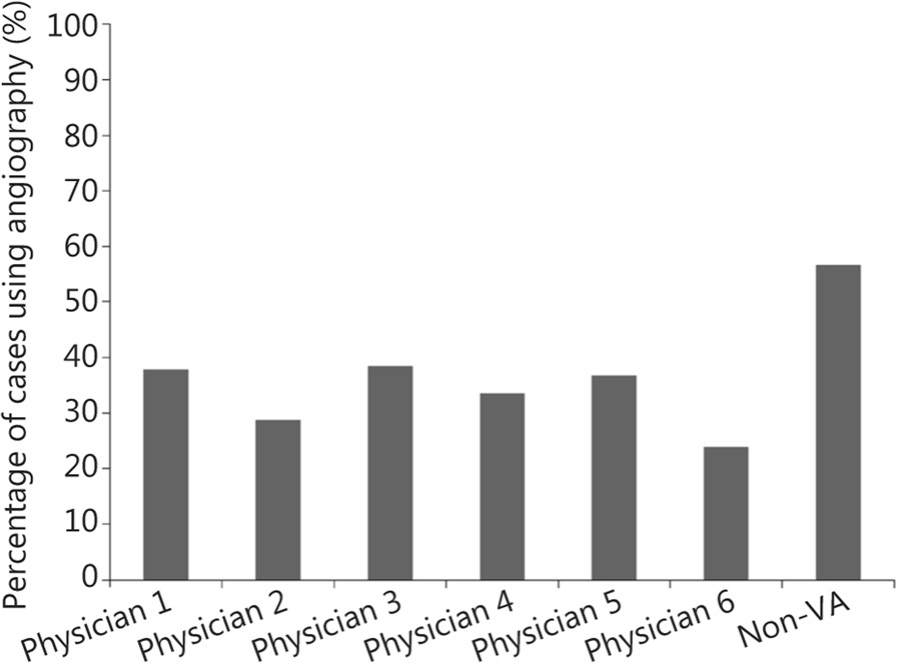
Difference in use of invasive coronary angiography between different physicians. Height of each bar represents the proportion patients with elevated troponin seen by a given provider (X axis) whose treatment plan included invasive coronary angiography

**Fig. 2:**
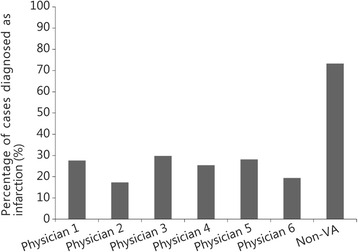
Difference in diagnosis of myocardial infarction between different physicians. Height of each bar represents the proportion of patients with elevated troponin seen by a given provider (X axis) whose diagnosis was myocardial infarction

A total of 761 patients were included. Patient characteristics and their clinical course were compared between the different physicians. Occasionally (i.e., weekends and holidays) inpatient services were provided by physicians from our affiliated academic medical center. Patients seen by these physicians were grouped under “non-VA Physician” (Table 1). While not randomly assigned, patient characteristics of the cohorts seen by each of the physicians were similar, except for chest pain (*P*<0.01). MI was diagnosed in 34.0 % of patients with positive Tn and in 25.9 % patients who underwent ICA. The rates of diagnosing MI (*P* = 0.02) and ICA use (*P* < 0.0001) differed between physicians, ranging from 23.9 to 56.7 % and 17.3 to 73.3 %, respectively (Figs. 1 and 2).

**Table 1:** Patient characteristics separated by each physician (%)

Characteristics	Physician 1 (*n* = 30)	Physician 2 (*n* = 29)	Physician 3 (*n* = 226)	Physician 4 (*n* = 218)	Physician 5 (*n* = 134)	Physician 6 (*n* = 57)	Non-VA Physician (*n* = 67)	*P*
Male	100.0	98.7	99.1	99.3	100.0	98.5	100.0	0.940
CAD	55.2	47.3	53.2	57.5	42.1	41.8	56.7	0.210
Hypertension	86.2	75.2	77.1	81.3	77.2	65.7	80.0	0.230
Diabetes	51.7	43.8	44.5	41.0	45.6	47.8	50.0	0.920
Hyperlipidemia	48.3	57.1	56.4	58.2	63.2	55.2	70.0	0.680
Smoking	10.3	10.2	8.3	11.2	3.5	7.5	23.3	0.110
Chest pain	27.6	23.5	32.1	22.4	29.8	19.4	53.3	0.005
Dyspnea	20.7	26.1	21.6	29.9	28.1	19.4	13.3	0.320
Abnormal ECG	31 %	18.1	16.5	13.4	19.3	14.9	26.7	0.270
New ECG changes	27.6	15.9	12.4	13.4	19.3	13.4	30.0	0.090
TIMI score >2	82.8	80.8	81.5	83.5	73.7	72.7	88.0	0.410
MI diagnosed	37.9	28.8	38.5	33.6	36.8	23.9	56.7	0.020
ICA done	27.6	17.3	29.8	25.4	28.1	19.4	73.3	<0.001

*CAD* Coronary artery disease, *ECG* Electrocardiogram, *ICA* Invasive coronary angiography, *MI* Myocardial infarction, *TIMI* Thrombolysis myocardial infarction

Multivariate regression for the diagnosis of MI was associated with the 1st Tn level (*OR* = 11.44, 95 % CI 4.60-28.48, *P* < 0.0001), chest pain (*OR* = 4.25, 95 % CI 2.84–6.37, *P* < 0.0001), and new ECG changes (*OR* = 3.88, 95 % CI 2.34–6.43, *P* < 0.0001). Creatinine was associated with a lower likelihood of MI diagnosis (*OR* = 0.68, 95 % CI 0.57–0.81, *P* < 0.0001) (Table 2). None of the physicians were independently associated with the diagnosis of MI. In multivariate regression, cardiac ICA was associated with several factors (Table 3), including chest pain (*OR* = 8.60, 95 % CI 5.2–14.2, *P* < 0.0001), thrombolysis in myocardial infarction (TIMI) score greater than 2 (*OR* = 3.27, 95 % CI 1.64–6.52, *P* = 0.001), abnormal ECG (*OR* = 1.89, 95 % CI 1.13–3.13, *P* = 0.014), and the 1st Tn level (*OR* = 1.71, 95 % CI 1.19–2.47, *P* = 0.004). Increasing creatinine levels were associated with a lower likelihood of ICA (*OR* = 0.70, 95 % CI 0.58–0.85, *P* < 0.0001). Physician 2 was associated with a lower likelihood of ICA (*OR* = 0.56, 95 % CI 0.34–0.90, *P* = 0.017), while care by a non-VA physician was more likely to result in ICA (*OR* = 4.45, 95 % CI 1.54–12.89, *P* = 0.006).

**Table 2:** Logistic regression showing variables associated with diagnosis of MI

Variable	β	S.E.	Wald	*P*	*OR*	95 % CI	
1st troponin level	2.437	0.465	27.426	<0.0001	11.44	4.60	28.48
Chest Pain	1.447	0.206	49.102	<0.0001	4.25	2.84	6.37
New ECG changes	1.356	0.258	27.675	<0.0001	3.88	2.34	6.43
Smoker	1.249	0.322	15.023	<0.0001	3.49	1.85	6.56
TIMI score > 2	1.248	0.298	17.579	<0.0001	3.48	1.94	6.25
Creatinine	−0.390	0.088	19.476	<0.0001	0.68	0.57	0.81
Constant	−2.247	0.323	48.451	<0.0001	0.106		

*CI* Confidence interval, *ECG* Electrocardiogram, *OR* Odds ratio, *SE* Standard error, *TIMI* Thrombolysis in myocardial infarction

**Table 3:** Logistic regression of variables associated with use of cardiac catheterization

Variable	β	S.E.	Wald	*P*	*OR*	95 % CI	
Chest Pain	2.152	0.257	70.079	<0.0001	8.60	5.20	14.23
Non-VA physician	1.493	0.543	7.563	0.006	4.45	1.54	12.89
TIMI > 2	1.185	0.352	11.317	0.001	3.27	1.64	6.52
Smoker	0.854	0.334	6.543	0.011	2.35	1.22	4.52
Abnormal ECG	0.634	0.259	5.986	0.014	1.89	1.13	3.13
Dyspnea	0.582	0.290	4.028	0.045	1.79	1.01	3.16
1st troponin level	0.539	0.185	8.441	0.004	1.71	1.19	2.47
Age (by year)	−0.031	0.011	8.780	0.003	0.97	0.95	0.99
Creatinine	−0.352	0.097	13.276	<0.0001	0.70	0.58	0.85
Physician 2	−0.590	0.248	5.654	0.017	0.56	0.34	0.90
Constant	−0.500	0.801	0.389	0.533	0.607		

*CI* Confidence interval, *ECG* Electrocardiogram, *OR* Odds ratio, *SE* Standard error, *TIMI* Thrombolysis in myocardial infarction

## Discussion

In this investigation, we demonstrated that the diagnosis of MI and the use of ICA vary by individual cardiology physicians within a single facility. After adjusting for differences in patient characteristics, physician-level variation in ICA use persisted. Because this was not a randomized trial and our sample size of providers was not adequate for hierarchical modeling, we cannot be certain that the observed variation was due to differences in the patient panel or to clinical decisions made by individual providers. For example, the non-VA physician category saw more patients with chest pain, a strong predictor of ICA use. Conversely, in our logistic regression model, we did not observe any differences between individual physicians with regard to the diagnosis of MI. While no formal definition of MI was applied, this would suggest that our physicians did use similar diagnostic criteria.

The overall rate of MI diagnosis Dx (34.0 %) and the use of ICA (25.9 %) were both low in this population despite the high risk of cardiovascular events. Approximately half the patients had prior CAD and diabetes, and a substantial majority had TIMI scores greater than 2. This observation is due, at least in part, to what appears to be widespread use among patients without typical symptoms of MI. Our observed rate of MI was even lower than the rate observed by Alcalai et al. [15]. We cannot accurately ascertain the reasons for this observation from our database. Given the high proportion of pre-existing CAD, type 2 myocardial infarctions or chronic, baseline elevation in Tn are plausible explanations. We did note in our logistic regression that an increased creatinine was associated with lower likelihood of both MI and ICA. Presumably, our physicians included elevated creatinine in their consideration of whether Tn elevation was due to MI. This also likely played a role in decisions concerning ICA use where consideration for avoiding iodinated contrast and preserving renal function are important factors.

In our analysis of ICA use, physician-level variation persisted within our regression model. One VA physician was associated with a lower likelihood of ICA perhaps reflecting a more conservative approach to MI management. The non-VA physician group had a higher propensity for ICA. This group was comprised of multiple physicians who do not practice regularly at our VA facility. We cannot determine if the difference in ICA use represents overuse by the non-VA physicians or underuse by those working at the VA. It is possible that the non-VA physicians, unfamiliar with a veteran population, felt that a more aggressive/invasive management strategy was warranted. VA physicians appeared to have similar rates of ICA use although this could represent widespread underuse.

The landscape of cardiology practice has changed substantially since the data for this investigation were first collected. The “troponin team” that once existed at our institution is no longer in use. The primary reason for its discontinuation was the high cost of the program (which involved tracking all patients with an elevated Tn and physician staffing). Furthermore, patient care was not substantially improved (i.e., short-term mortality tracked in the facility was not changed with the use of the troponin team). We no longer have physicians who share part-time staffing duties. All physicians are now full-time VA employees. As such, we do not have any immediate plans to alter our patterns of care based on the results of our investigation.

Our data were gathered as part of a clinical demonstration project and not primarily as a research investigation. As a result, definitions of MI and decisions to perform ICA were not standardized. Patients were not randomly assigned, and residual differences in patient populations after logistic regression may have occurred. A multi-level model controlling for patient characteristics within each physician cohort would have been a preferable statistical approach; however, our sample of physicians was too small for this to be a valid approach. This limited sample size and the blinding of the identities of individual physicians precludes any analysis based on subspecialty within cardiology. The strongest variation by an “individual physician” in our investigation was actually the group of non-VA physicians with too few encounters to analyze individually, limiting the ability to compare our data with other investigations of individual physician care variations.

## Conclusion

The likelihood of a patient with an elevated Tn being diagnosed with MI and undergoing ICA is dependent on their clinical presentation and may possibly depend on the responsible physician. Whether this variation represents overuse or underuse is unclear.

## Abbreviations

CI, confidence interval; ECG, electrocardiogram; ICA, invasive coronary angiography; MI, myocardial infarction; OR, Odds ratio; TIMI, Thrombolysis in myocardial infarction; Tn, Troponin; VA, Veterans affairs
